# Intelligence, Cognition, and Language of Green Plants

**DOI:** 10.3389/fpsyg.2016.00588

**Published:** 2016-04-26

**Authors:** Anthony Trewavas

**Affiliations:** Institute of Molecular Plant Science, University of EdinburghEdinburgh, UK

**Keywords:** behavior, fitness, intelligence, signals, assessment, game theory, self-organization

## Abstract

A summary definition of some 70 descriptions of intelligence provides a definition for all other organisms including plants that stresses fitness. Barbara McClintock, a plant biologist, posed the notion of the ‘thoughtful cell’ in her Nobel prize address. The systems structure necessary for a thoughtful cell is revealed by comparison of the interactome and connectome. The plant root cap, a group of some 200 cells that act holistically in responding to numerous signals, likely possesses a similar systems structure agreeing with Darwin’s description of acting like the brain of a lower organism. Intelligent behavior requires assessment of different choices and taking the beneficial one. Decisions are constantly required to optimize the plant phenotype to a dynamic environment and the cambium is the assessing tissue diverting more or removing resources from different shoot and root branches through manipulation of vascular elements. Environmental awareness likely indicates consciousness. Spontaneity in plant behavior, ability to count to five and error correction indicate intention. Volatile organic compounds are used as signals in plant interactions and being complex in composition may be the equivalent of language accounting for self and alien recognition by individual plants. Game theory describes competitive interactions. Interactive and intelligent outcomes emerge from application of various games between plants themselves and interactions with microbes. Behavior profiting from experience, another simple definition of intelligence, requires both learning and memory and is indicated in the priming of herbivory, disease and abiotic stresses.

## What is Intelligence?

The word intelligence derives from the Latin *intelegere*; to choose between. In situations of choice if the decision made after assessment is beneficial, it is considered to be an intelligent decision. Legg and Hutter (2007) collected some 70 different definitions of intelligence and summarized them as follows. Intelligence: (i) Is a property that an individual has as it interacts with its environment or environments. (ii) Is related to the agents ability to succeed or profit with respect to some goal or objective. (iii) Depends on how able the agent is to adapt to different objectives or environments.

In the same numerical order. (i) Wild plants interact with and respond to their environment via competitive and other biotic and abiotic signals. (ii) The goal or objective is fitness with seed number as a fitness proxy. Those most successful, and thus most fit, provide more offspring. (iii) Fitness depends on the skill with which individuals best adapt to their environment throughout their life cycle (McNamara and Houston, 1996). Those individual plants that can master and adapt to the problems of competition, master other biotic and abiotic stresses with greater plasticity, lower cost, higher probability, or more rapidly, are fitter and on this basis are more intelligent. Finally intelligence is a capacity for problem solving, (the psychologists choice) and profiting from experience another (Jennings, 1923; Gardner, 1983; Sternberg and Detterman, 1986; Sternberg, 1986). All effectively say the same thing.

### Intelligent Behavior Requires Information Processing

Communication is fundamental to the induction and control of behavior. Whether between molecules, between cells, tissues and organs or within the greater system that encompasses the external environment, the release of information, its interpretation and return to indicate receipt, enable development to continue and change in a continual dynamic throughout the life cycle. The bit is the standard unit of information, a yes/no answer and some attempts have been made to estimate bits of information involved for some cellular signal transduction processes (Trewavas, 2014). So far the range of estimates indicates few outcomes to the received information. At a more complex level, cell-to-cell or tissue-to-tissue, information that is released requires an information return to indicate not only its receipt but also its correct interpretation to ensure the processes of behavior continue in a fashion that provides for a fitter organism. The accuracy with which information is gathered from the environment, from other cells and from the short- and long-term memories in that cell, how it is used or rejected or stored for later use and other information sent back is a primary challenge but one that currently has not been taken up by plant biologists. Yet its determination is fundamental to understanding behavior, intelligence, and fitness. The complexity of signaling within the plant described later indicates the scale of the problem.

## Mcclintock’s Thoughtful Cell

McClintock (1984), a plant biologist, was awarded the Nobel Prize. In her acceptance speech she stated “*a goal for the future would be to determine the extent of knowledge the cell has of itself and how it uses that knowledge in a thoughtful manner when challenged.*” The response to ‘challenge’ is behavior and ‘thoughtful’ responses are intelligent behavior. McClintock’s linking ‘cell’ with ‘thoughtful’ was a far-sighted appreciation of the actual capabilities of the cell. The capability emerges when comparisons are made with systems known to act in intelligent fashion; that is, nervous systems.

### Cells and Nervous Systems are Complex Systems

A system is a network of mutually dependent and thus, interconnected components comprising a unified whole. It is the connections and characteristics of these connections or interactions that, determines the ultimate property of any system. Many natural systems and indeed, some man-made ones are extremely complex. The formation of any system requires an exchange of information between the interacting constituents that reflects the accuracy of fit between them. The more exact the fit the higher the level of information involved in the interaction. Those systems of concern to biology are dynamic; the constituents change and/or strength of interactions are modified enabling the construction of different channels of information flow to accommodate and interpret differing circumstances and signals (Trewavas, 2014).

Cells and nervous systems are self-organizing complex networks that act holistically. The cell system is constructed from an estimated 100,000 different cellular protein species that act as both linking and computational elements to form an interactome (Bray, 1995). These links ensure the cytoplasm of plant cells can be centrifuged down leaving an enzyme-free supernatant (Kaempner and Miller, 1968). A simple nervous system is that of *Caenorhabditis elegans*, a nematode worm, containing some 300 or so neurons to form a connectome. Anatomical study and single neuron ablation studies has provided potential functions of each. An optimized wiring network has been deduced (Gushchin and Tang, 2015).

Empirical data and analytical models of many complex networks have shown that connection patterns in many real networks, including cells and nematodes, converge to a similar architecture exhibiting a heterogeneous degree (degree = connection or link) between the components with the distribution characterized by a power law with a minority of highly connected nodes. These systems have therefore both a core and periphery distinguished on their degree; hub species have lots of degrees and connectors few. This arrangement provides for the interpretation of numerous signals and pathways of information flow and thus engenders resilience (Gao et al., 2016).

Both cells and nematodes respond to extracellular signals. Nematodes process volatile and water soluble chemicals, touch, osmolarity, etc., using sensory cells connected to sensory neurons, amplification via interneurons where assessment is made and thence to motor neurons which excite different kinds of muscle (Hobert, 2003; Chatterjee and Sinha, 2008). Behavior is modified by experience via non-associative and associative learning through adaptation, habituation, and decision capabilities when response has to be prioritized between two contrasting signals (Hobert, 2003; Giles and Rankin, 2009). Cells process information (when responding to external signals) through receptor activation, amplification (through cytosolic Ca^2+^, G proteins, numerous protein kinases) and then to a motor output involving ion flux, gene expression and movement in those single cells capable of it.

### Systems Structure Similarity between Interactome and Connectome

Detailing the interactome requires numerous high quality interaction maps avoiding inevitable false positives (Yu et al., 2008). The interactome of *Homo sapiens* covering about a third of potential proteins, has an average degree of about 7; yeast with more than three quarters of all proteins examined has an average degree of 10 (Wuchty, 2014). Degrees are distributed on a power law. In the nematode connectome, the average degree is again about 7 and the distribution of degrees is a power law with a minority of neurons with very high degrees of connection and a long tail down to 3–4 notably in the posterior of the animal (Hall and Russell, 1991; Chatterjee and Sinha, 2008; Varshney et al., 2011).

In the cytoplasm, high degree proteins are in combination with large numbers of others, e.g., actin that is thought to combine with upward of 100. If eliminated by mutation, these hubs are usually found essential for growth and division and described as a lethality-centrality principle (Wuchty and Almaas, 2005; Zotenko et al., 2008). High-degree proteins are involved in control of the whole network (Liu et al., 2011). A Minimum Dominating Set (MD Set) has been defined as optimized subsets of proteins from where each protein in the subset may be immediately reached. MD Set modules control network behavior (Nacher and Akutsu, 2012; Wuchty, 2014). Not all MD Set proteins are high degree. Wuchty (2014) provides a toy model of degree number in an MD Set. In both yeast and humans these MD Set proteins are about one sixth of the total protein number; their average degree increased from 7 to 17 in *H. sapiens* and in yeast, from 10 to 24. They do contain proteins implicated in the development of cancer and in viral infection. The normal behavior of the cell is overcome in these situations.

In the *C. elegans* connectome a ‘rich club’ of neurons with degree 44 and above are found in some 11–12 neurons (Varshney et al., 2011; Towlson et al., 2013). Ablation of most of these, affects locomotion and it is considered that they represent core command and assessment neurones required to integrate signals. They are connector hubs, highly connected to each other and with many inter-modular connections and thus to nodes in different modules. The equivalent in an MD Set to these rich club neurons, might be enzymes, e.g., protein kinase C with an estimated degree of 50. Beitkrutz et al. (2010) show that the phosphorylome is densely interconnected arising from cross-phosphorylation between numerous protein kinases including MAP kinases. The MD Set does contain numerous protein kinases.

When the connectome learns, information flow is altered by changing connection weight; increasing or decreasing synaptic number or by plasticity in synaptic strength. In that way a pathway through which information flows is deepened and pathway speed elevated. In cellular interactomes signal transduction pathways involving changes in cytosolic Ca^2+^ and the subsequent cellular cascade are responsible (Gee and Oertner, 2016). The interactome learns through the initial cytosolic Ca^2+^ changes, subsequent amplifying transduction cascades and numerous protein kinases changing again information flow. This pathway will last usually as long as the new protein phosphorylation state remains. Entirely new pathways can also be constructed through novel tertiary structure changes in transduction proteins involving different phosphorylation sites and control (Trewavas, 2014).

The motor elements are usually to be found in changing ion flux and protein complement and the transduction trace acts as memory. Networks that control their own information flow are intelligent (Vertosick, 2002). Both the connectome and the interactome exhibit intelligent capabilities.

### McClintocks Thoughtful Cell: Can Single Cells be Conscious?

Margulis and Sagan (1995) described consciousness as basically ‘*awareness of the outside world’*. Any organism that exhibits sensory perception and responds to it has some awareness of its outside world (Trewavas and Baluska, 2011). But awareness of a world outside of itself also requires awareness of itself, i.e., self-recognition. *C. elegans* is aware of the outside world and uses it to profit from experience but so does *Physarum polycephalum*, a single but multinucleate cell. This coenocyte learns to anticipate simple repetitive signals, such as three electrical or touch cues, by momentary reductions in growth rate. It responds to the expected but un-provided fourth signal by equivalent growth rate reductions (Saigusa et al., 2008). There are many similar capabilities expressed by other single cells (Trewavas, 2014). *Physarum* recognizes itself because when any of its growth lobes meet, growth usually ceases; it will not try to exploit different branches of itself. Any network as complex as the interactome and connectome, will be conscious and if we ever build an artificial network with this kind of structure, it will likely be conscious too.

An alternative more limited definition of consciousness is the use of mental images to regulate behavior. Because animals have always had to move to find food, predation of animal on animal evolution refined sensory and motor equipment and joined the two with a rapid connection and later assessment system of nerve cells compacted into a brain. The ancestors of plants having acquired photosynthesis found, beneficially, light energy ubiquitously distributed but to contain the osmotically active products required a relatively rigid wall enormously inhibiting individual movement. The individual plant containing many millions of cells is a self-organizing, complex system with distributed control permitting local environmental exploitation but in the context of the whole plant system. Consciousness is thus not localized but is shared throughout the plant in contrast to the more centralized location in the animal brain. But from a system framework, the real plant is the individual together with its environmental connections and its self-constructing niche (Trewavas, 2014).

### The Root Cap Entity

The root cap is an example of plant consciousness or awareness. It is located at the extreme tip of the root. It is a dynamic structure of about 200 cells consisting of the extreme tip and surrounding peripheral cells and a central columella in three vertical stories of about 48 cells that do, under some conditions, containing gravity sensing statoliths (See Blancaflor et al., 1998 for diagrammatic representations). The cap both senses and assesses numerous signals: gravity using statoliths; touch which initiates an unusual dog-leg kind of structure in the distal growing region placing the tip at an angle enabling the tip to slide over an obstacle surface (Massa and Gilroy, 2003); phosphate deficiency, signals are transmitted to the shoot which synthesizes novel sRNA’s fundamentally changing root morphology (Svistoonoff et al., 2007); soil nitrate gradients causing an acceleration of growth along the gradient and cessation when rich sources are encountered (McNickle and Cahill, 2009); humidity gradients, when water is in short supply, statoliths are dismembered preventing interference by gravity signals (Eapen et al., 2003); salt stress which initiates long distance cytosolic Ca^2+^ waves to the shoot (Choi et al., 2014). The response motor for all these signals is located shoot-wards in the elongating region as a result of information transmission from the cap first reported by Darwin (1880).

Root cap cell ablation has been examined only with gravity signals (Blancaflor et al., 1998). Different cell groups have differential inputs to five different gravi-response parameters. Effect of loss varies from zero to nearly 90% of response loss and indicating that about only 10% of the columella cells are the most critical. In the *C. elegans* connectome about 4% are critical. A central controlling core of root cap cells (with high degree and high connectivity) surrounded by a less significant periphery (with low degree and lower connectivity) is indicated and the necessary controlling structure for intelligent behavior. Mechanical signals are initiated via cytosolic Ca^2+^ transients in the peripheral cells (Massa and Gilroy, 2003). Although the root cap acts holistically, different sensory and assessment functions are distributed amongst different cell types.

Darwin (1880) first described the root tip as behaving like the brain of a lower animal. The analogy is most certainly valid but without the involvement of any nerve cells in plants.

## Intelligent Behavior in the Plant Kingdom

If single cells can act intelligently, how much more so when dealing with plants containing billions of cells.

Behavior is defined as the response to signals. Without muscular movement, visible behavior in plants is usually the result of changes in growth. But growth is slow, well below our animal capacity to see it without deliberate measurement. And yet the initial changes in plant cells after signaling, either as action potentials or changes in cytoplasmic Ca^2+^ occur at speeds little different to those in animals. The present categories of known signals, whose change in strength elicits discrete changes in plant growth pattern (phenotype) and molecular alteration, are light, temperature, mechanical stimuli, water, gravity, soil structure, electrical changes, numerous chemicals (volatile and non-volatile), and biological signals. The changes in development are attempts to construct a phenotype that best optimizes the fitness possibilities within the framework of the present and future environmental context (Trewavas, 2014).

### Plants are Self-organizing Organisms

There are many examples of self-organization in biology and two typical ones are social insect colonies (swarm intelligence) and the mammalian brain. Plants typically develop by a kind of Markovian series; there is no overall plan, it is the interactions that generate order from the bottom up rather than top down from the first fertilized cell onward (Trewavas, 2014). Trees are typically self-organizing consisting of millions of repetitions of modular structures, leaf, plus bud above ground and below ground, branch root tips. Flexibility results from being able to marshal large numbers of modules toward necessary objectives. The tree is a complex network, as are all plants, in which fairly simple rules of interaction qualitatively change with size.

### Situations Requiring Decision and Choice

Growing tissues in any plant compete with each other. This phenomenon of competitive correlation is easily shown by removal of one and observing enhanced or accelerated growth of others. There is competition between stem branches, between root and shoot, between roots, between vegetative and reproductive structures, between different fruits, between flower buds, between seeds, between fruit and flowers, between vegetative buds, and between the apical bud and others, (Sinnott, 1960). The control of competition for resources must rely on internal signaling. Electrical signaling and waves of free cytosolic Ca^2+^ traverse long distances (Bose, 1906; Choi et al., 2014; Xiong et al., 2014). These early changes are reinforced by changed circulating signals of proteins, peptides, small, and large RNAs (mRNAs), oligosaccharides, hormones, natural pesticides, volatile chemicals, other growth modifying chemicals as well as various carbohydrates, minerals, and water. The plant is more like a giant cell with the complexity of information that is transported.

To help optimize fitness, the plant phenotype is modified to best extract external resources. Tremmel and Bazzaz (1993), illustrate how this process works out in competitive circumstances. The cambium, a meristem, that structurally is like a kind of inner skin in both root and shoot, is the motor tissue involved in this process of assessment, decision, and choice. Comparative assessments are continually made of the productivity of different branches and alterations made in the number of vascular elements. Productive branches receive more; less productive have some blocked and very poor are completely blocked off and die. The dynamic is continuous throughout the life cycle requiring a running commentary as it were of comparative return, finely balanced choice and decision (Sachs et al., 1993; Sachs, 2006). Manipulating the extent of vascular connection in this way can explain much of the observed competitive aspect within individual plants between what are called sources and sinks, between vegetative and reproductive tissues and even between different fruits although the discrimination there may depend on the closeness of the newly hybridized genome to that of the mother plant (Trewavas, 2014).

Leaves on many trees and shrubs from sub-tropical to the boreal maintain a temperature of 21.4 ± 2.2°C during the growing season and a temperature optimal for photosynthesis (Helliker and Richter, 2008). Homeostasis can be maintained through a variety of short-term manipulations, stomatal control, cell chloroplast, and leaf blade movement and, slightly longer term, hair number, cuticular wax reflectance modifications and leaf numbers on branches. There are numerous choices between different combinations of these but what makes the decisions, the homeostatic controller is not known. Stomatal behavior is optimized by formation of dynamic leaf patches that form from automaton systems of epidermal leaf cells that interact via mechanical signaling (Trewavas, 2014).

### Spontaneity, Error Correction, and Counting to Five or More Indicate Intention in Plant Behavior

Behavioral spontaneity in any organism implies that it possesses the capability to control its own behavior and thus its own information flow. Spontaneity can be recognized when organisms and/or cells behave differently from others in the same circumstances. The evidence for spontaneity in plants is reasonably extensive but it has remained unrecognized, because behavior is usually expressed as an average, thus eliminating individual behavior. Individuality in gravitropic responses has been detailed (Bennet-Clerk and Ball, 1951; Selker and Sievers, 1987; Ishikawa et al., 1991; Zieschang and Sievers, 1991). In addition when placed with the seed root vertically upward only 25% grew vertically downward; the remainder grew in all different directions (Ma and Hasenstein, 2006). An upward vertical direction is not a very sensitive stimulus for gravitropic responses. Roots do learn about gravity signals and then construct the equipment to more sensitively respond. Other spontaneity is exhibited by situations in which an increasing strength of signal results in more tissues or cells responding and seed germination is a typical example of many others (Bradford and Trewavas, 1994; Trewavas, 2012).

The ability to correct errors in behavior indicates that a plant possesses internal information as to the expected course of events. It indicates awareness of what is right and ‘knows’ when it is going wrong. The Venus fly trap (*Dionea*), an insectivore, contains two hairs that must be touched within 20 s of each other for the trap to close. Five touches, induced by continual prey movement, elicits secretion of digestive enzymes, sodium channel formation, and prey digestion that takes many weeks. By default one, three, and four touch stimuli are recognized (Bohm et al., 2016). Trap triggering can be elicited experimentally with a needle or by very small unsuitable prey, but the trap opens again within a day. Similarly, for *Drosera*, the sundew; when insects land on the sticky tentacles, other tentacles bend to touch and trap the insect further and then the whole leaf envelops the prey and digests it over many weeks. Charles Darwin showed that pieces of inert material, chalk or a small piece of stone, cause some initial bending of the tentacles but these then rapidly straighten, resetting the trap.

Climbing plants using tendrils can be provided with a support and winding is initiated. If the support is removed, the tendril unwinds, straightens, and seeks new supports elsewhere. This can be conducted numerous times before the tendril habituates. Unsuitable supports, a glass rod for example, initiate winding. But the tendril then again unwinds straightens and searches elsewhere (Trewavas, 2014). Tendrils will accommodate their shape to any surface they contact initially but usually undo that shape and search elsewhere. Similar observations have been made with plants that climb through stem winding. If they contact a support and start to wind and then the support is removed, the stem will straighten and the search continued elsewhere. Supports are recognized and plants move toward them (Trewavas, 2005).

## Volatile Relationships and Games Between Friends and Aliens

One offshoot of the profuse photosynthetic fixation of carbon is the synthesis of an enormous range of volatile organic chemicals (VOCs) emitted by roots, shoots, leaves, bark, fruits, and flowers. When shoots are attacked by herbivores or disease organisms the spectrum of emitted volatiles change and that can attract parasitoids of the herbivorous pests (so-called burglar alarm). Functions for some of these, like methyl jasmonate, ethylene, and methyl salicylate, are known and these three are involved in the induction of defense mechanisms (Paré and Tumlinson, 1999; Dudareva et al., 2006). If plants are grown close together, then adjacent, un-attacked plants then emitted VOCs can also initiate defense mechanisms providing they are within about 50 cm in wind-still conditions such as are more frequently found in forests than on open ground (Karban, 2008; Heil and Adame-Alvarez, 2010). The lack of species specificity of this process raises questions as to its real biological value because the normal interactions between individuals are usually regarded as competitive not cooperative, although some exceptions are occasionally reported. A potential, perhaps better, function of herbivore-induced volatile production is to overcome some limitations of the vascular system since not all areas of the plant are equally connected together with regions damaged by herbivores, (Holopainen and Blande, 2012).

### VOCs Act as the Plant Language

The VOC spectrum is different between individual species and even individuals, (Dudareva et al., 2006). VOCs account for 1% loss of fixed carbon and obvious fitness benefits arise from those emitted by flowers and fruits. But relevance in other tissues can be less obvious. Holopainen and Blande (2012) have creatively suggested that the complexity and species individuality of VOC act as a plant vocabulary or language; individual volatiles are words and the VOC signature represent sentences. In a strict sense, a sentence is an emergent property of the words used to construct it (Trewavas, 2014). If equivalent, it suggests that the whole VOC signature due to synergy between the words is essential; omission of one or two words will fail, something now reported. The whole VOC signature not individual volatiles are required to elicit insecticidal responses (Kikuta et al., 2011). The VOCs emitted by damaged shoots elicit greater response in genetically identical relatives than aliens even from the same species suggesting the potential for self-recognition and perhaps altruism (Karban and Shiojiri, 2009; Karban et al., 2013). Spontaneity suggests each individual will likely emit its own signature. But closely grown plants also exhibit the shade avoidance syndrome growing away from individual competitors because competition between individuals is the basis of Darwinian evolution (Trewavas, 2014).

That plants can sense alien volatiles is known. The young seedlings of Dodder, a parasitic plant, home in on their prey by sensing the direction of emitted volatiles (Runyon et al., 2006). Even with mature dodder seeking new hosts, half of all contacts with potential prey are rejected after a few hours and new prey sought elsewhere. The initial contact is with the stem or bark surface both of which emit their own volatile signature, reflecting their current health and nutrient status. If the prey is accepted, the likely energy return from a new host is assessed within just those few hours and the total energy to be used for parasitism (assessed as a number of coils), is calculated (Kelly, 1992). The assessment of coil number indicates again a potential ability to count and for a larger number than five. The number of coils determines the numbers of haustoria which are formed only after several days when coiling is complete.

There are numerous reports that indicate that volatiles emitted by rhizosphere bacteria and mycorrhizae alter root architecture either in branching, enhanced growth of main root or root hair production by VOCs (Gutierrez-Luna et al., 2010; Bitas et al., 2013; Castulo-Rubio et al., 2015; Ditengou et al., 2015, and references therein). Thus, there has to be root sensing mechanisms and receptor proteins present. If alien species of plant root emit these volatiles they will induce root proliferation too.

However, one other potential use of volatiles occurs in leaf mimicry and sensing of unsuitable trees for climbing. *Boquilia trifoliolata*, a climbing vine in temperate rainforests, mimics the leaves of its supporting hosts in terms of size, shape, color, orientation, petiole length, and/or tip spininess. The mimicry has the beneficial effect of reducing herbivory and thus increasing fitness. So far Gianoli and Carrasco-Urra (2014) have reported mimicry on at least eight different hosts and most crucially a vine, extending across different hosts, responds to each in turn. Sensing and action on particular released host bark VOCs is the most likely mechanism here. It is also known that some vines simply avoid trees on which the trunk is too smooth to enable climbing (Trewavas, 2014). Again VOC recognition explains this phenomenon.

What is suggested here is that the VOC signature is used as information below ground, not only to discourage potential predators like nematodes, but also to provide information about root identity and crucially self-recognition. The range of volatile chemicals produced below ground is quite extraordinary (e.g., Rasmann and Turlings, 2008; Ens et al., 2009; Palma et al., 2012; Fiers et al., 2013; Musah et al., 2016) and sufficient to account for the complexity of self and alien recognition which is known to occur. There have been claims that root exudates can distinguish self/non-self plants. But despite the ease with which such chemicals could be analyzed, no chemical with the required self-recognition properties has been reported (Semchenko et al., 2014).

How are VOCs sensed? Since plants synthesize many VOC’s they do have enzymes with active sites that produce the chemical in the first place and thus have the potential with slight modification of producing a similar protein for sensing them. To simplify the detection of the VOC signature a single protein receptor detecting only partial structures of all the individual VOC signature complex is indicated by the information above (Kikuta et al., 2011). This is known as odotope theory.

### The Players of Games

Game theory, originally constructed to study economic competition, was later adopted to understand behavioral interactions between animals. But it has found considerable use in plant behavior studies in particular in respect to competition (Trewavas, 2014). Its value lies in the predictions made for certain kinds of game since they identify the measurements necessary to indicate the specific characteristics of competition. Competition requires recognition of self and of those aliens that enter the game. The quoted references contain the necessary mathematics of interaction.

Tit-for-tat occurs in plants rooted in the same soil who recognize each other as aliens and proliferate but leaving a clear zone between the two (Schenk et al., 1999; Gersani et al., 2001). The detection of the alien root system is most likely through emitted VOCs or other diffusible chemicals in the soil as indicated above. Crucially, morphological changes depend more on the identity of competitive neighbors than local resource distribution thus indicating again ability to distinguish self from aliens (Caldwell, 1996; Huber-Sannwald et al., 1997).

Further demonstrations of self and alien recognition have used plants constructed with two shoots and two roots and placed in various combinations adjacent to each other with alternating self and alien root systems adjacent (Falik et al., 2003). When aliens are placed adjacent to each other, root proliferation results. These experiments have been taken further by vertically separating the two shoot and two root into separate plants and growing them apart. After several months the clones, originally part of the same plant, now react as though they are alien to each other (Gruntmann and Novoplansky, 2004). It is the VOC signature that best explains these observations because in each individual plant there will be a drift in the expression of particular proteins due to noise in the control circuitry and a very high sensitivity in response to even slight environmental variation (Trewavas, 2014).

The Prisoners Dilemma is a game in which collaborative activity produces greater overall benefit; but that individual cheating on the collaboration benefits the individual more. Symbiotic relations with nitrogen-fixing rhizobia in nodules and mycorrhizal fungi that penetrate root cells and provide phosphate, exemplify the game. Cheaters are recognized in both situations, rhizobial bacteria taking carbohydrate but providing little N and in mycorrhizae fungal hyphae accumulating phosphate for themselves and not passing it on. In the former, the host increases the oxygen content of the nodule reducing the fitness of the rhizobium. Mycorrhizal cheats may cause host-defense reactions or hosts insist on a strict one-to-one molecule exchange of carbohydrate for phosphate.

Mycorrhizae form extensive mycelial networks and can connect together separate plants. Surprisingly, information on disease and herbivory can pass through this network to other host network partners enabling them to prepare defense reactions (Trewavas, 2014; Gorzelak et al., 2015).

## Profiting and Priming from Experience

### Priming Indicates that Experience is Learnt and Changes Behavior

Those plants that experience herbivory or disease become primed to further insults so that they now respond more quickly, to a greater extent and thus more robustly, than unchallenged plants (Van Hulten et al., 2006; Ton et al., 2007; Frost et al., 2008). Priming can last for years and in certain cases survives meiosis. Chromatin structural modification, through epigenetic changes (specific histone acetylation or phosphorylation, DNA methylation), are the probable basis (Jaskiewicz et al., 2011; Singh et al., 2014).

But priming is now recognized to occur after repetitive heat, drought, cold and salt stresses which train the plant to respond more quickly and more robustly to these conditions (Ackerson, 1980; Ding et al., 2012; Sani et al., 2013). The experience is learnt and remembered; that memory participates in subsequent experiences. This learnt experience has now altered subsequent behavior in ways that will impact on fitness. Repetitive treatments with the hormone, abscisic acid (ABA), primes ABA dependent genes in the same way (Goh et al., 2003); their expression now responds more quickly and to a greater extent to subsequent hormone treatments.

Repetitive signaling mimics the process of learning in animals and establishes a memory that is incorporated into subsequent signaling processes. Gagliano et al. (2014) have used the well-known leaf folding response of *Mimosa pudica* to mechanical signals to demonstrate that repetitive stimulation institutes an habituation memory that lasts several hours. Alternative mechanical stimulation demonstrated that habituation was not the result of sensory adaptation.

Perhaps more intriguing is the obvious cross talk between many of these abiotic stressful conditions in which some of the same events are induced by separate stresses. Thus the response to one like heat helps resistance to cold stress (Cheong et al., 2002; Rizhsky et al., 2004). Similarly, herbivory attack increases resistance to disease (Koorneef and Pieterse, 2008). These observations represent kinds of conditioned behavior in which one signal influences response to another and increase fitness. They are analogous to the distribution of function and cross reactions in complex brains. The life history of individual cloned plants determines their capability for stress response and priming illustrating how sensitive plants are to slight environmental variation (Raj et al., 2011).

Stress induced signal-transduction involves information flow through calcium-dependent processes and protein kinase pathways. Concomitantly, synthesis of the constituents of these pathways are increased, deepening the metabolic channel through which information flows (Trewavas, 1999, 2014). During brain learning synaptic connections are strengthened and/or new connections made thereby deepening the channel of information flow through particular neural pathways. Although short-term memory in the brain involves glutamate sensitive channels, longer term involves modification of the nerve cells themselves and similar epigenetic changes could be involved. The potential for plant cells to learn new transduction pathways arises from new phosphorylation sites exposed when transduction proteins undergo uncommon conformational shifts (Trewavas, 2014).

## Author Contributions

The author confirms being the sole contributor of this work and approved it for publication.

## Conflict of Interest Statement

The author declares that the research was conducted in the absence of any commercial or financial relationships that could be construed as a potential conflict of interest.
